# Growth and Selective Etch of Phosphorus-Doped Silicon/Silicon–Germanium Multilayers Structures for Vertical Transistors Application

**DOI:** 10.1186/s11671-020-03456-0

**Published:** 2020-12-09

**Authors:** Chen Li, Hongxiao Lin, Junjie Li, Xiaogen Yin, Yongkui Zhang, Zhenzhen Kong, Guilei Wang, Huilong Zhu, Henry H. Radamson

**Affiliations:** 1 Key Laboratory of Microelectronic Devices and Integrated Technology, Institute of Microelectronics, Chinese Academy of Sciences, Beijing, 100029 People’s Republic of China; 2 University of Chinese Academy of Sciences, Beijing, 100049 People’s Republic of China; 3 Research and Development Center of Optoelectronic Hybrid IC, Guangdong Greater Bay Area Institute of Integrated Circuit and System, Guangdong, 510535 China

**Keywords:** Phosphorus-doped silicon, SiGe, RPCVD, Dopant segregation, Auto-doping, Selective etch

## Abstract

Vertical gate-all-around field-effect transistors (vGAAFETs) are considered as the potential candidates to replace FinFETs for advanced integrated circuit manufacturing technology at/beyond 3-nm technology node. A multilayer (ML) of Si/SiGe/Si is commonly grown and processed to form vertical transistors. In this work, the P-incorporation in Si/SiGe/Si and vertical etching of these MLs followed by selective etching SiGe in lateral direction to form structures for vGAAFET have been studied. Several strategies were proposed for the epitaxy such as hydrogen purging to deplete the access of P atoms on Si surface, and/or inserting a Si or Si_0.93_Ge_0.07_ spacers on both sides of P-doped Si layers, and substituting SiH_4_ by SiH_2_Cl_2_ (DCS). Experimental results showed that the segregation and auto-doping could also be relieved by adding 7% Ge to P-doped Si. The structure had good lattice quality and almost had no strain relaxation. The selective etching between P-doped Si (or P-doped Si_0.93_Ge_0.07_) and SiGe was also discussed by using wet and dry etching. The performance and selectivity of different etching methods were also compared. This paper provides knowledge of how to deal with the challenges or difficulties of epitaxy and etching of n-type layers in vertical GAAFETs structure.

## Introduction

As the scaling of complementary metal oxide semiconductor (CMOS) reaches its physical limitation, the short-channel effects significantly weaken the performance of transistor. A solution to these problems is new transistor designs, e.g., GAAFETs (gate-all-around field-effect transistors), which is also considered as the most promising candidate for nanoscale transistors down to 3-nm technology node [1–6]. Lateral and vertical nanowires/nanosheets are main structures according to International Roadmap for Device and Systems (IRDS) 2020 to replace FinFETs [7]. Vertical GAAFETs (or vGAAFETs) have free flexibility design on gate length and have great potential to increase integrated density [4, 8]. There are two main categories to implement vertical nanowire structures: bottom-up and top-down. The bottom-up method introduced metal catalyst, which may bring process compatibility issues [9, 10]. The top-down method is the mainstream in the industry because of its better control of nanowire configuration and its compatibility with FinFET [4, 11–14]. The top-down method to fabricate vertical GAAFETs attracts much attention. Self-alignment gate with accurate gate-length control was a crucial issue [15, 16]. To achieve better effective gate length control or reduce variation, the gate length could be primarily determined by the thickness of the channel material epitaxially grown on a bottom flat surface, such as Si/SiGe/Si, and SiGe was the channel material [17–20]. Moreover, another critical integration challenge lies in the doping between channel and S/D regions [16, 20, 21], especially with sharp junction control [20]. Compared with the traditional source/drain implantation process, epitaxy process simplifies the fabrication process, reduces surface damage, and achieves uniform doping profile. However, the P-doped Si/SiGe/P-doped Si sandwich structure is difficult to grow epitaxially due to the segregation, auto-doping and out-diffusion phenomena of the most common n-type dopants, phosphorus [22, 23], arsenic [24, 25], and/or antimony [26, 27] at Si/SiGe interface in chemical vapor deposition (CVD) systems. Therefore, the segregated donor atoms gathered at the Si/SiGe interface and the SiGe layer would be doped, which may degrade the transistor performance with high leakage.

One strategy to impede the dopant segregation is to apply very low growth temperature. There are a series reports to make many efforts to grow n-type doping by molecular beam epitaxy (MBE) [28]; meanwhile, this method has not been applied for CVD. MBE equipment is mostly single-chip design, requiring high vacuum and slow throughput. Moreover, MBE equipment is not compatible with wafer sizes above 8 inches in industry. Therefore, MBE technology is not suitable for industrial mass production applications. However, RPCVD system has strong production capacity and simple equipment structure, which is suitable for IC industry [29]. The first idea for RPCVD growth is to regulate the hydrogen flow in the chamber since the hydrogen is the carrier gas and can affect the kinetics of precursor gases. Li et al*.* [23] reported that hydrogen changed the bonding structure of host atoms in the surface and reduced the segregation energy by applying low growth temperature in rapid thermal CVD (RTCVD) system. However, the effect of hydrogen has not been explored at Si/SiGe interface in reduced pressure CVD (RPCVD) system. Suvar et al*.* [30] inserted 30-nm undoped Si spacer layers between P-doped Si and SiGe to lower the P concentration at the interface by a factor of 4 (from 8 × 10^19^ cm^−3^ to 2 × 10^19^ cm^−3^), but the P doping peak cannot be eliminated. Bennett et al*.* [31] have studied the effect of strain on n-type doping in Si. The solid solubility of doping was increased by introducing tensile strain in Si. Christensen et al*.* [32] have found no significant dependence of the P diffusivity on the Ge content in Si_1-x_Ge_x_ (0 ≤ x ≤ 0.22). And the P diffusion coefficients had little difference between relaxed Si and biaxially compressive-strained SiGe. Zangenberg et al*.* [33] observed an enhancement of diffusion coefficient by a factor of 2 at 825 °C for relaxed Si_0.88_Ge_0.12_.

In this paper, several methods have been proposed to improve P incorporation in Si in a multilayer of Si/SiGe/Si using RPCVD. In the experiments, different strategies such as hydrogen purge, inserting undoped spacer layers, changing the Si precursor from SiH_4_ to SiH_2_Cl_2_ (DCS), and modulating the strain profile by introducing Si_0.93_Ge_0.07_ sacrificial layer on both sides of SiGe layer have been presented. Furthermore, the selective etch of SiGe was discussed to form thin SiGe layer (intended as channel layer) [6, 34]. The etching characteristics of wet and dry etching tools were also compared. The final structure is intended to be used for vGAAFETs for sub-10-nm technology node in the future.

## Methods

Si/SiGe/Si multilayers (MLs) were grown on 200-mm Si <100> wafers with RPCVD (ASM Epsilon 2000) equipment. The Si substrates were cleaned with mixture solution of H_2_SO_4_ and H_2_O_2_, followed by diluted HF to remove native oxide prior to inserting into the load locks of epitaxy chamber. The samples were in situ cleaned by annealing at 1050 °C to remove the native oxide to obtain high-quality surface of Si. The precursors for the Si, Ge, and P were SiH_4_ (or SiH_2_Cl_2_), 10% GeH_4_ in H_2_, and 2% PH_3_ in H_2_. The growth temperature was 650 °C, while the chamber pressure was kept at 80 Torr during epitaxy. In some experiments, the chamber pressure was reduced to 10 Torr to grow P-doped Si_0.93_Ge_0.07_ layer in the source/drain (S/D) regions. The Ge content in the SiGe channel was kept constant to 0.22. To study the selective etching characteristics, 50 nm nitride/30 nm oxide was deposited as hardmask to protect the nether MLs. Lithography and dry anisotropic vertical etch were performed to form separate cuboid patterns. Selective etch experiments were carried out with wet etch tool of HF (6%):H_2_O_2_ (30%):CH_3_COOH (99.8%) = 1:2:4 and dry etch tool of CF_4_:O_2_:He = 4:1:5 [35].

The Si/SiGe/Si MLs were characterized by the techniques of high-resolution (Thermo Scientific Talos F200) transmission electron microscopy (HRTEM), energy-dispersive X-ray spectroscopy (EDX), high-resolution X-ray diffraction (HRXRD), and high-resolution reciprocal lattice map (HRRLM) from Bruker JV Delta-x, scanning electron microscopy (SEM) from Hitachi (Japan), and secondary ion mass spectroscopy (SIMS).

## Results and Discussion

### Epitaxy of P-Doped Si/SiGe/Si MLs

**Fig. 1:**
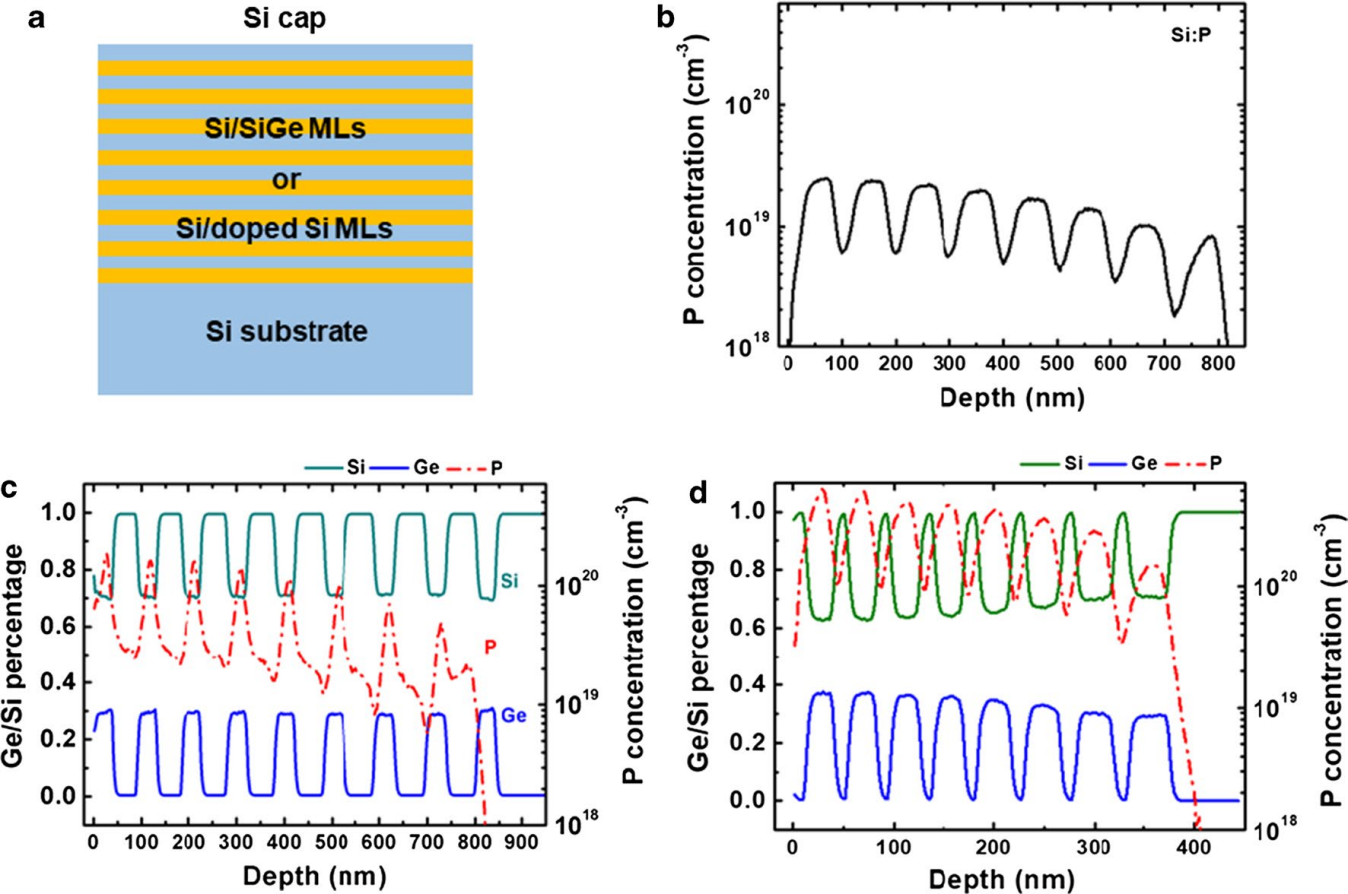
**a** Schematic diagram of P-doped Si/SiGe/Si MLs. **b** P-doping concentration of undoped Si/P-doped Si MLs. Ge/Si percentage and P concentration of **c** undoped Si_0.72_Ge_0.28_/P-doped Si, **d** undoped Si/P-doped Si_0.72_Ge_0.28_ MLs. No purging and undoped spacer layer were considered

In this study, the incorporation of P in Si and SiGe was initially explored. The ML structures are shown in Fig. 1a. A ML of P-doped Si/undoped Si with increasing PH_3_ flow was grown, and the layer profiles were examined by SIMS in Fig. 1b. The figure shows P concentration increases and reaches the highest level of 2.6 × 10^19^ cm^−3^. Two more samples with profile of ‘P-doped Si/Si_0.72_Ge_0.28_/P-doped Si’ and ‘Si/P-doped Si_0.72_Ge_0.28_/Si’ were designed, and the P-profile is demonstrated in Fig. 1c, d, respectively. In Fig. 1c, a P pile-up is observed at interfaces of P-doped Si/Si_0.72_Ge_0.28_ multilayers. The interfacial P pile-up increases with increasing P concentration from the bottom to the top in the multilayers, and the highest concentration is 1.6 × 10^20^ cm^−3^, which is 6 times as much as the concentration in Fig. 1b (2.6 × 10^19^ cm^−3^). In doped Si_0.72_Ge_0.28_ layers (Fig. 1d), P concentrations are remarkably higher, and there are no peaks at the interface. Because of doping, the Ge percentage is slightly increased. This behavior is related to the enhanced adsorption of SiH_4_ and GeH_4_ in the presence of PH_3_. Besides, due to doping, the layer thicknesses are different between Fig. 1c, d, which have the same growth time. It means that P-doping enhances the growth rate of Si_0.72_Ge_0.28_ layers and the absorption of GeH_4_, while the growth rate of Si is retarded due to P adsorption. These phenomena are consistent with the outcome reported in Refs. [36–38]. From the above, P segregation and auto-doping phenomenon are serious at Si/SiGe interface. The P-doping peak at the Si/SiGe interface makes unintentional doping in the SiGe layer. Since SiGe is intended as the channel layer in the transistors, the inhomogeneous doping profile or high background doping levels would limit device applications [39]. Several methods to eliminate the P peak would be discussed below. For better comparison, all SiGe layers are strained, and the flow ratio of SiH_4_ (SiH_2_Cl_2_) and GeH_4_ for the SiGe layer was not altered throughout all the experiments.

### Impact of Spacer Layers

**Fig. 2:**
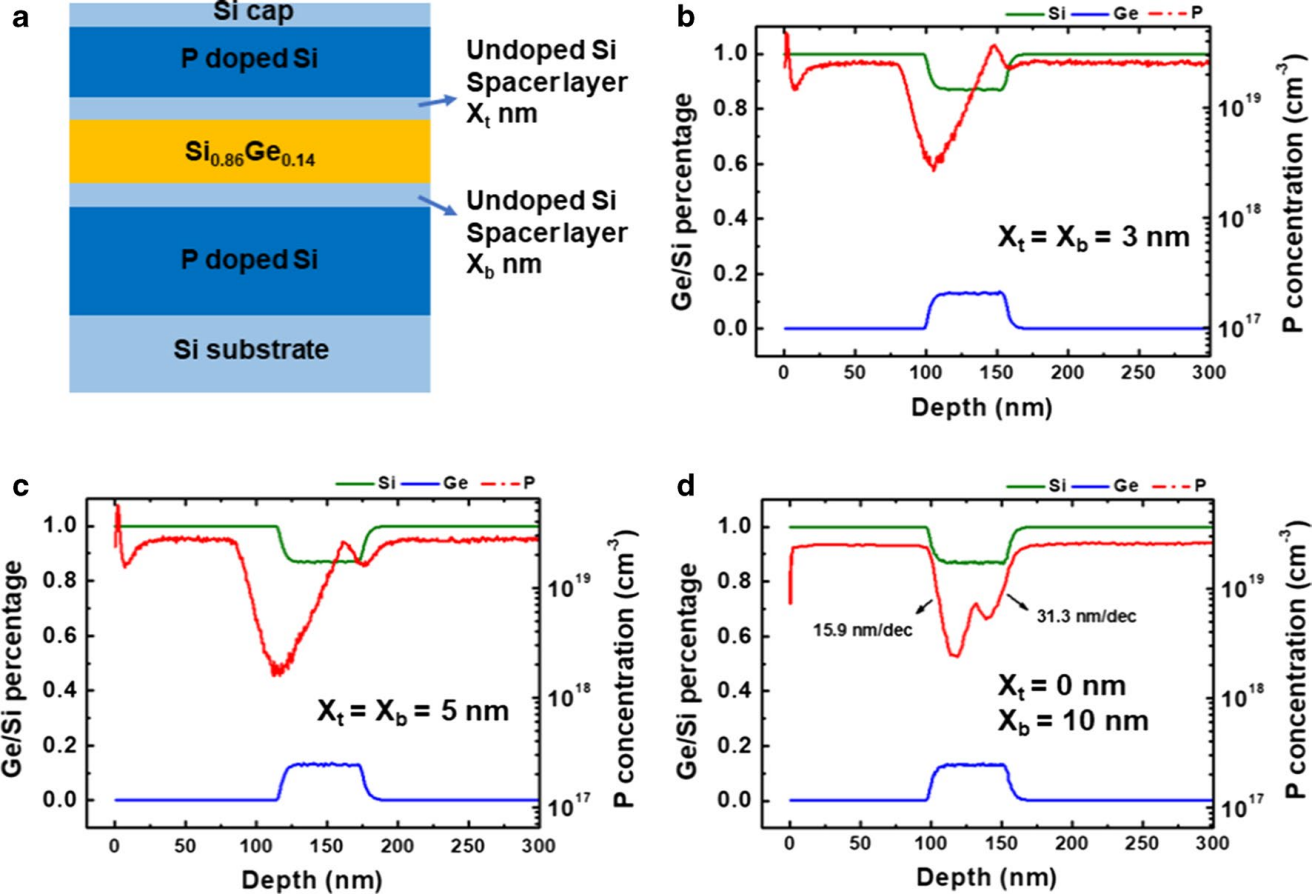
**a** The schematic of experimental samples with different undoped spacer layers. And Ge, Si, and P profiles of P-doped Si/Si_0.86_Ge_0.14_/P-doped Si MLs with undoped Si spacer layers of **b** 3 nm, in both interfaces, **c** 5 nm, in both interfaces, **d** 10 nm, only at one interface with Si_0.86_Ge_0.14_

**Fig. 3:**
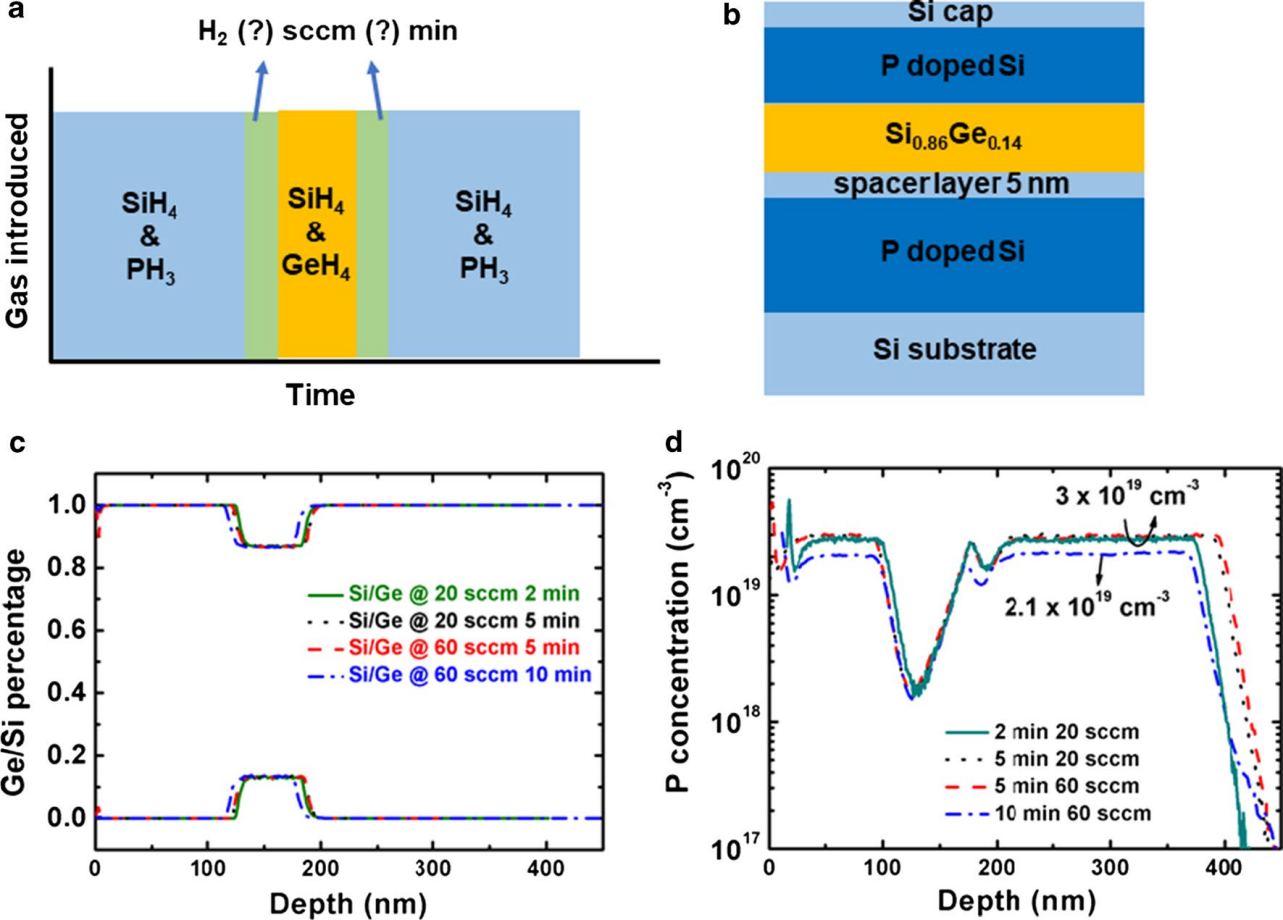
Schematic diagrams of **a** doping strategy of H_2_ purge conditions, and **b** experimental structure of Si/SiGe/Si MLs. **c** Ge/Si profile and **d** P concentration of P-doped Si/Si_0.86_Ge_0.14_/P-doped Si MLs

**Fig. 4:**
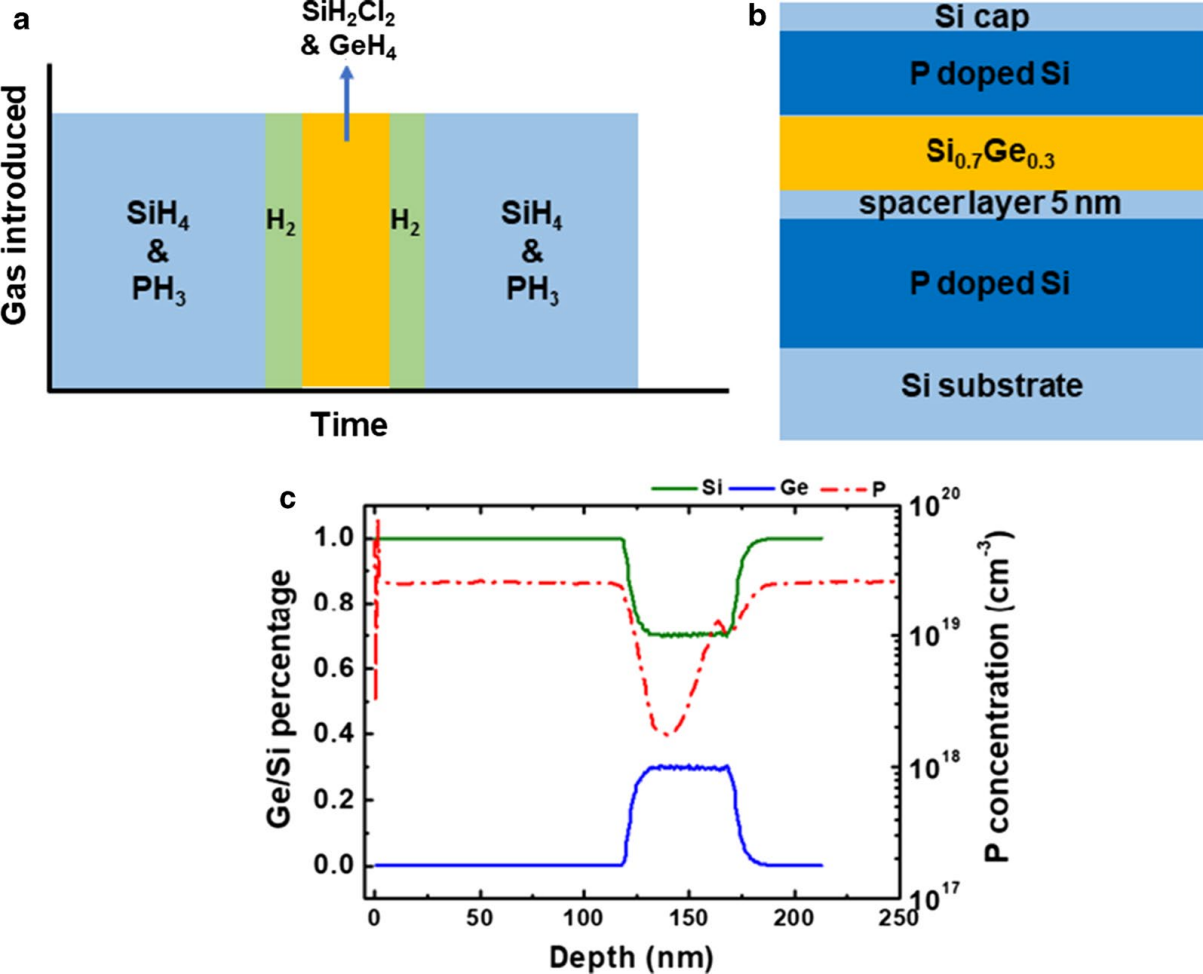
Schematic diagrams of **a** doping strategy of changing growth chemistry, **b** experimental structure of Si/SiGe/Si MLs. The SiGe layer was grown with DCS. The purge time was 5 min with flow of 60 sccm after doped Si. The undoped Si spacer layer was 5 nm between bottom-doped Si and undoped SiGe. **c** Ge/Si profile and P concentration of P-doped Si/Si_0.7_Ge_0.3_/P-doped Si MLs

**Fig. 5:**
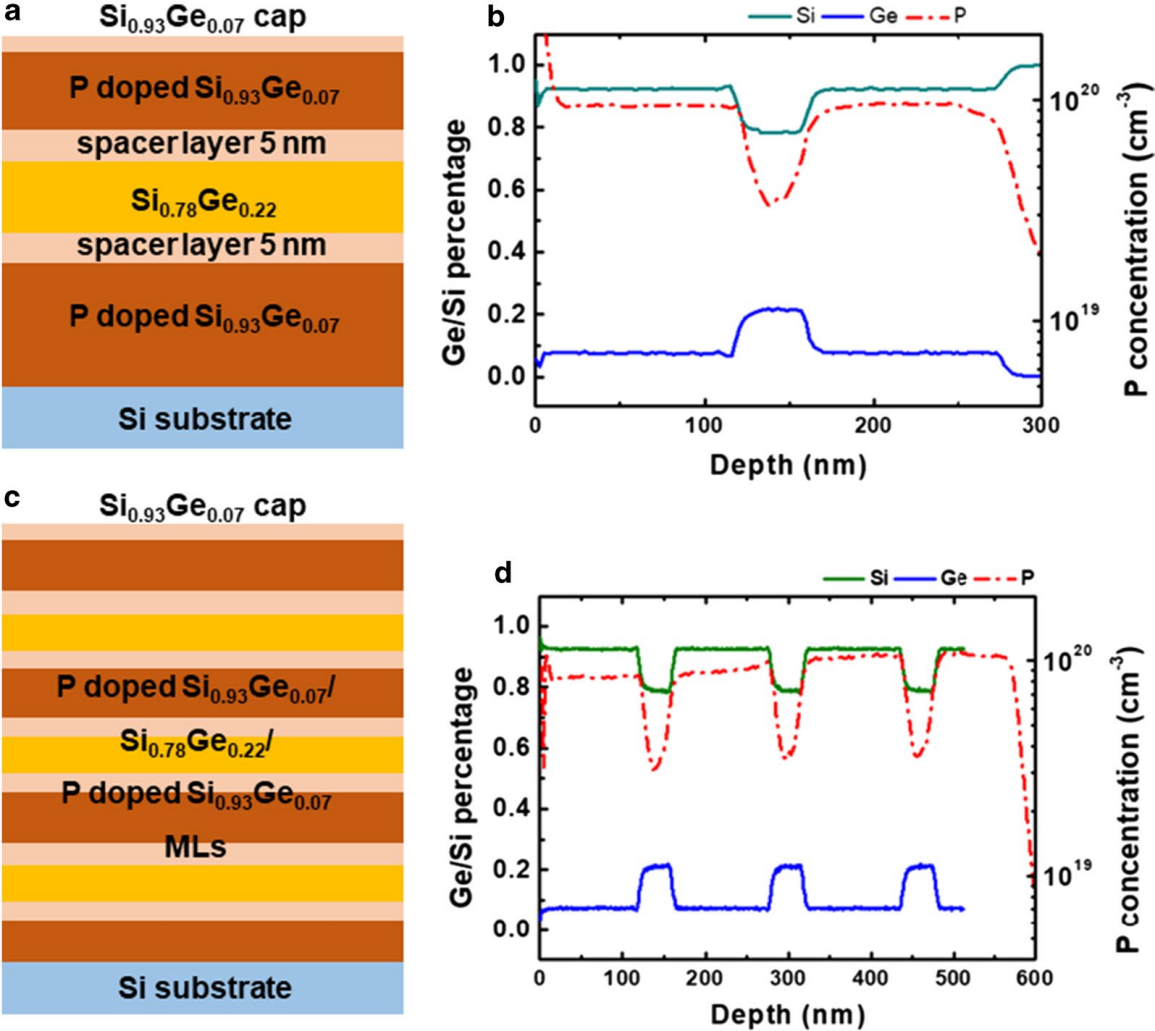
**a** Schematic diagram, **b** Ge/Si and P profile in one layer of P-doped Si_0.93_Ge_0.07_/Si_0.78_Ge_0.22_/P-doped Si_0.93_Ge_0.07_ ML. **c** Schematic diagram, **d** Ge/Si and P profile in three layers of P-doped Si_0.93_Ge_0.07_/Si_0.78_Ge_0.22_/P-doped Si_0.93_Ge_0.07_ ML

**Fig. 6:**
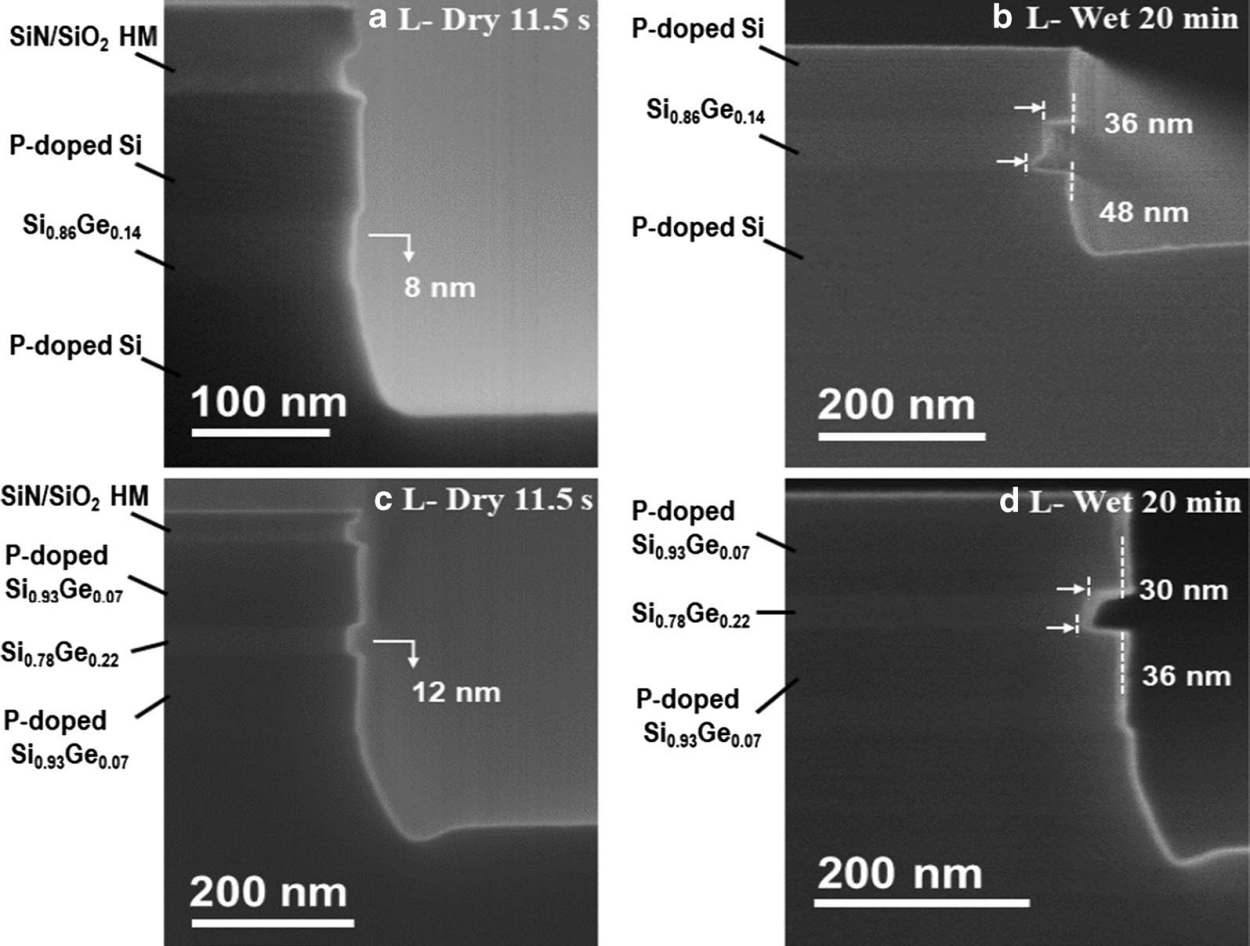
SEM images of P-doped Si/Si_0.86_Ge_0.14_/P-doped Si in Fig. 2c with **a** 11.5-s dry etch, **b** 20-min wet etch, and P-doped Si_0.93_Ge_0.07_/Si_0.78_Ge_0.22_/P-doped Si_0.93_Ge_0.07_ MLs with **c** 11.5-s dry etch, **d** 20-min wet etch. The dry etch was CF_4_:O_2_:He = 4:1:5, and the wet etch was HF (6%):H_2_O_2_ (30%):CH_3_COOH (99.8%) = 1:2:4

**Fig. 7:**
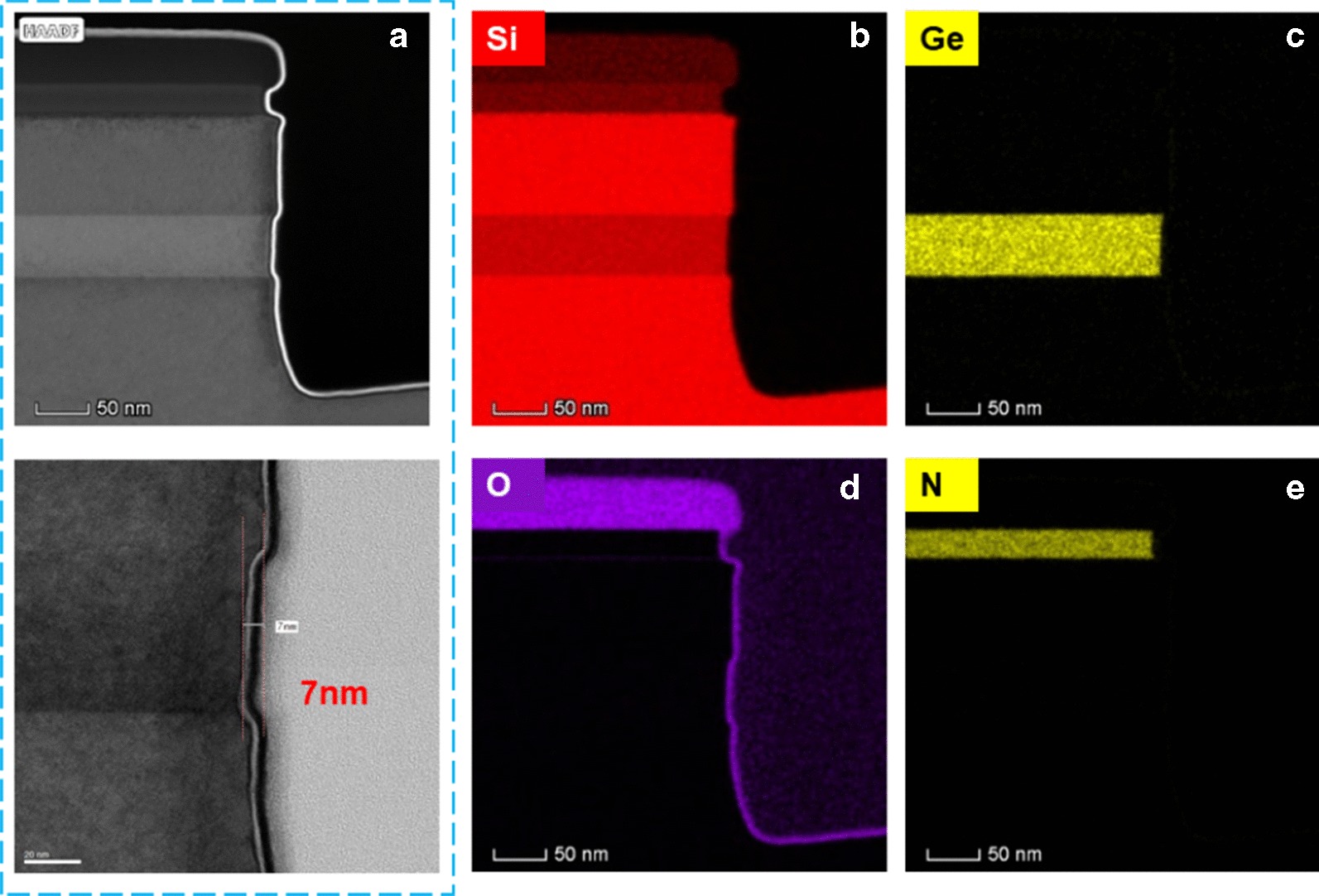
**a** TEM images, **b**–**e** EDS mapping of P-doped Si/Si_0.86_Ge_0.14_/P-doped Si in Fig. 6a with 11.5-s dry etch. The elements in **b** is Si, in **c** is Ge, in **d** is O, and in **e** is N

Undoped Si spacer layers were inserted between the bottom-doped Si layer and undoped SiGe layer to absorb the excess of P atoms. Figure 2a shows the schematic diagram of the designed structure, and Fig. 2b–d demonstrates the profile results from integrated Si spacers with thickness of (b) 3 nm, (c) 5 nm, and (d) 10 nm. The peaks of P pile-up are reduced, while the Si/Ge percentage and P concentration in Si layers are kept constant as in Fig. 2b–d. The P pile-up level is reduced by 82%, from 4 × 10^19^ cm^−3^ in Fig. 2b to 7 × 10^18^ cm^−3^ in Fig. 2d, when the spacer thickness *X*_b_ increased from 3 to 10 nm. Increasing the thickness of undoped Si spacer layers increases the absorption of excessive P atoms. In Fig. 2d, the slope of P-profile at Si_0.86_Ge_0.14_/Si surface is 15.9 nm/dec, while at Si/Si_0.86_Ge_0.14_ interface the slope is 31.3 nm/dec. Meanwhile, too thick Si spacer layer is not an appropriate solution since the sheet resistance increases. Therefore, a trade-off between sheet resistance and uncontrolled of P-profile has to be made for transistors. Figure 2 reveals also the impact of spacer layer between the Si/Si_0.86_Ge_0.14_ layers (*X*_b_) was different from the layer between the Si_0.86_Ge_0.14_/Si (*X*_t_). In Fig. 2b, c, the spacer thicknesses between the Si_0.86_Ge_0.14_/Si were 3 nm and 5 nm, while in Fig. 2d, no spacer layer was inserted. However, the slope of P-profile at the Si_0.86_Ge_0.14_/Si is the same (about 15.9 nm/dec), although in Fig. 2d the top spacer layer was removed but no influence on the doping profile was observed. From the above results, the P peak was only at the Si/Si_0.86_Ge_0.14_ interface, which was possibly due to the solubility limit; the excess of P atoms may form P–P dimers at surface and be incorporated in the SiGe cap layer. Moreover, there is an auto-doping of P during the SiGe growth after P-doped Si. Therefore, the methods to clear the excess of P atoms or improve the Si solubility have been sought.

### Impact of Hydrogen Purge at the Interface of Si/SiGe/Si MLs

In this section, Si spacer layer was fixed at 5 nm, and hydrogen purge was introduced to clear the excess of P atoms after the P-doped Si growth. It can be seen from Fig. 3c, d that increasing the hydrogen flow from 20 to 60 sccm and purge time from 2 to 10 min has no obvious effect on the P peak. The doping concentration in Si is 3 × 10^19^ cm^−3^, which is the same as discussed in section “Impact of Spacer Layers”. The P peak concentration at the interface is the same with concentration in Si from Fig. 3d. The layer thicknesses are the same under different purge conditions. The P atoms cannot be cleared by hydrogen; this can be explained by the formation of stable P complexes on the surface. By changing parameters such as temperature, pressure, purge time would be helpful [24, 40], but too long purge time is not suitable due to time cost, and high temperature (> 950 °C) causes Si-Ge interdiffusion [41].

### Impact of Growth Chemistry on P-incorporation

In these experiments, the Si precursor, SiH_4_, has been replaced to SiH_2_Cl_2_ (DCS). In these samples, the growth parameters were the same as before, and the structures contain 5-nm Si spacer layer and the purge time is 5 min with flow of 60 sccm. The idea behind is to investigate whether Cl-based chemistry could clear the excess P atoms by Si surface and reactions of P-Cl, Si-Cl or Ge-Cl could happen [42]. From Fig. 4, the P peak concentration reduces by a factor of 2 (from 2.6 × 10^19^ cm^−3^ to 1.3 × 10^19^ cm^−3^), and the P concentrations in Si layers are 2.6 × 10^19^ cm^−3^. The estimated Ge content is 30%, which is higher than SiGe with SiH_4_. The higher Ge content demonstrates that Cl removed preferably the Si atoms at the surface reactions. This result also can be explained by the different relationship of gas flow ratio and Ge concentration with SiH_4_ and SiH_2_Cl_2_ gaseous precursors [32, 43]. Another explanation was that Ge atoms increased hydrogen desorption, then increasing free nucleation sites [44]. The P concentration slope of the Si_0.7_Ge_0.3_/Si interface was 13.2 nm/dec, which was a little sharper than Si_0.86_Ge_0.14_/Si interface (15.9 nm/dec). The slope of P-profile at the Si/Si_0.7_Ge_0.3_ interface was 20 nm/dec. Therefore, by introducing more HCl or increasing the gas ratio of SiH_2_Cl_2_ and GeH_4_, the segregated P atoms at the doped Si surface can be etched by HCl to form P-Cl dimers and the P peak concentration at Si/SiGe might be lower [38, 45].

### Impact of Ge Content on P-profile

As we discussed before, the incorporation of P in SiGe was remarkably higher than in Si. Therefore, this may raise the idea of adding a few percentages of Ge (7%) in Si spacers (5 nm) could improve the incorporation of P in Si. It is worth mentioning here that our purpose is not to change significantly the character of P-doped Si but impede the segregation of P in Si. In these samples, chamber pressure reduced to 10 Torr during the growth of spacer layers. The doping-dependent growth rate and Ge percentage would be important on this condition. From Fig. 5b, the top and bottom layers were 110 nm Si_0.93_Ge_0.07_ with P concentration of 1 × 10^20^ cm^−3^, the middle layer were 40 nm Si_0.78_Ge_0.22_ with P concentration of 3.5 × 10^19^ cm^−3^. The P concentration slope of P-doped Si_0.93_Ge_0.07_/Si_0.78_Ge_0.22_ was about 33 nm/dec. The slope was not sharp because the Ge percentage difference between the two layers was not large enough. In Fig. 5d, three layers of P-doped Si_0.93_Ge_0.07_/Si_0.78_Ge_0.22_/P-doped Si_0.93_Ge_0.07_ MLs was grown to verify the doping uniformity, and its structure diagram was shown in Fig. 5c. It can be seen, from bottom to top layers, the P concentration was decreasing, which can be explained by the memory effect of P. The residual P atoms in the chamber or diffused P atoms accumulate at the film surface and block free active sites on the surface [38, 39]. Although the P-peak had been eliminated, the segregation between Si_0.78_Ge_0.22_ and Si_0.93_Ge_0.07_ was still serious.

### Selective Etching Characteristics of Si/SiGe/Si MLs

When the ML structure is successfully grown (using the above growth strategies), the NWs have formed by vertical etch using SiO_2_/SiN as hardmask. Afterward, SiGe layer has to be selectively etched to Si in the lateral direction to form the channel layer with a designed width. In these experiments, two types of ML structures have been chosen: P-doped Si/SiGe/P-doped Si (sample-1, in Fig. 2c) and P-doped Si_0.93_Ge_0.07_/Si_0.78_Ge_0.22_/P-doped Si_0.93_Ge_0.07_ (sample-2, in Fig. 5b). These choices are made according to above discussions where the out-diffusion of P has been (partially) suppressed, as well as the perspectives of device application are considered.

The etch in the vertical direction was performed by dry etch, while for lateral etch a selective dry or wet etching was applied. The etching profiles of sample-1 are shown in Fig. 6a, b. And the TEM image and EDS mapping of Fig. 6a has been shown in Fig. 7. In these experiments, the hardmask is oxide/nitride. Figure 6a shows after 11.5 s dry etching of CF_4_/O_2_/He. The etch selectivity of Si_0.86_Ge_0.14_ and P-doped Si is 5.8. Figure 6b shows that after 20 min wet etch of HF (6%)/H_2_O_2_ (30%)/CH_3_COOH (99.8%). The wet etch has removed the hardmask (SiO_2_/SiN), and as a result, Si cap layer was etched ~ 10 nm as well. As discussed in section “Impact of Spacer Layers”, there is a P pile-up at the P-doped Si/Si_0.86_Ge_0.14_ interface. The wet etch is sensitive to the doping level; therefore, the first interface was etched faster. As a result, the front etch interface is not vertical and it is faceted or angled. The average selectivity was less than 4.2. Comparing the two etching methods, dry etch is sensitive to Ge percentage with better selectivity of SiGe, while wet etch is sensitive to dopant concentration. The etchings of sample-2 are also studied in Fig. 6c, d. Similar phenomena were observed in this sample, while the SiGe selective etched depths were deeper (Fig. 6a, c) due to higher Ge percentage. In dry etch, the selectivity of Si_0.78_Ge_0.22_ and P-doped Si_0.93_Ge_0.07_ was 6.3, while in wet etch, the average selectivity was less than 2.5. Therefore, dry etch was a better choice in consideration of etching uniformity and selectivity.

**Fig. 8:**
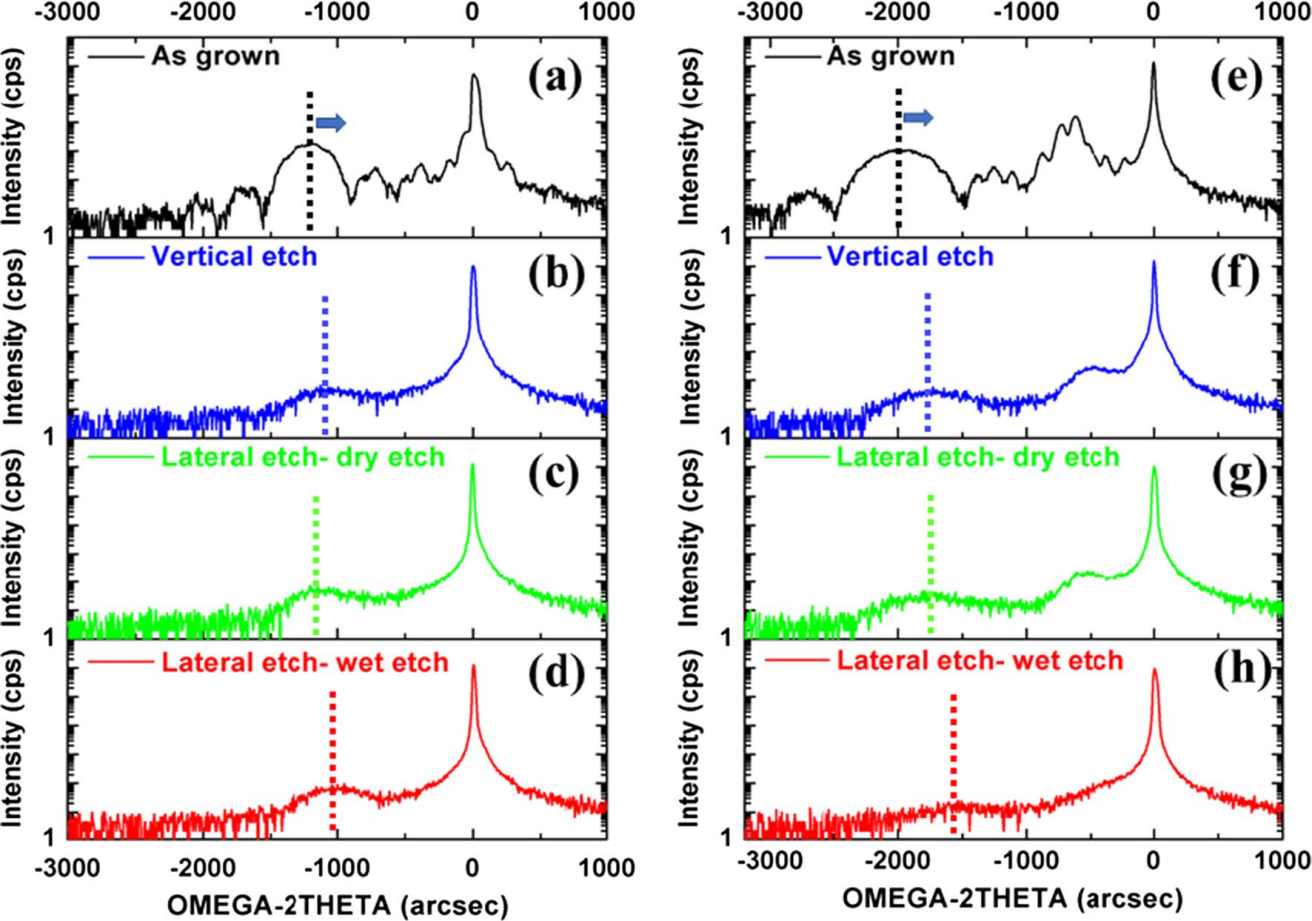
HRXRD rocking curve around (004) reflection of sample-1, P-doped Si/Si_0.86_Ge_0.14_/P-doped Si MLs with 5 nm spacer layer in **a**–**d**, and sample-2, P-doped Si_0.93_Ge_0.07_/Si_0.78_Ge_0.22_/P-doped Si_0.93_Ge_0.07_ MLs in **e**–**h**. Both the two samples have four panels: as grown, after vertical etch, SiGe lateral wet etch of HF (6%)/H_2_O_2_ (30%)/CH_3_COOH (99.8%) 20 min, and lateral dry etch of CF_4_/O_2_/He 11.5 s

Further analyses were performed to investigate the strain after etch steps in sample-1 and sample-2. Figure 8a–h shows (004) rocking curves (RCs) from these samples as follows: as-grown, after vertical etch, and SiGe lateral etch using wet and dry etching. In RC analysis, the broadening (full-width-half-maximum or FWHM) is an indicator for the defect density, and the position of SiGe peak compared to Si determines the strain amount in the layer. We emphasize here that the peak broadening can be also due to the thin thickness of the layer. Therefore, it will be difficult to distinguish from RC analysis the contribution of defect density, but we can only compare FWHM in some extensions in these analyses. In these RCs, sample-1 (Fig. 8a–d) has a single SiGe layer; meanwhile, sample-2 (Fig. 8e–h) shows two peaks representing 7% and 22% Ge. For As-grown samples, an interference of X-ray beam is observed, which causes thickness layer fringes. The emerging of these fringes shows a high-quality SiGe/Si interface. In RCs, of sample-1 and sample-2, the Ge peak has shifted toward the Si substrate peak indicating strain relaxation. No further shift of Ge peak has been detected after lateral dry etch of SiGe. This is a promising outcome for the transistor performance since the carrier mobility in the channel region is dependent on the strain. Meanwhile, the strain has been more relaxed for the wet-etched SiGe, and more shift toward the substrate peak has been observed. This shows that the wet etch is not suitable for the lateral SiGe etch, forming the channel layer.

**Fig. 9:**
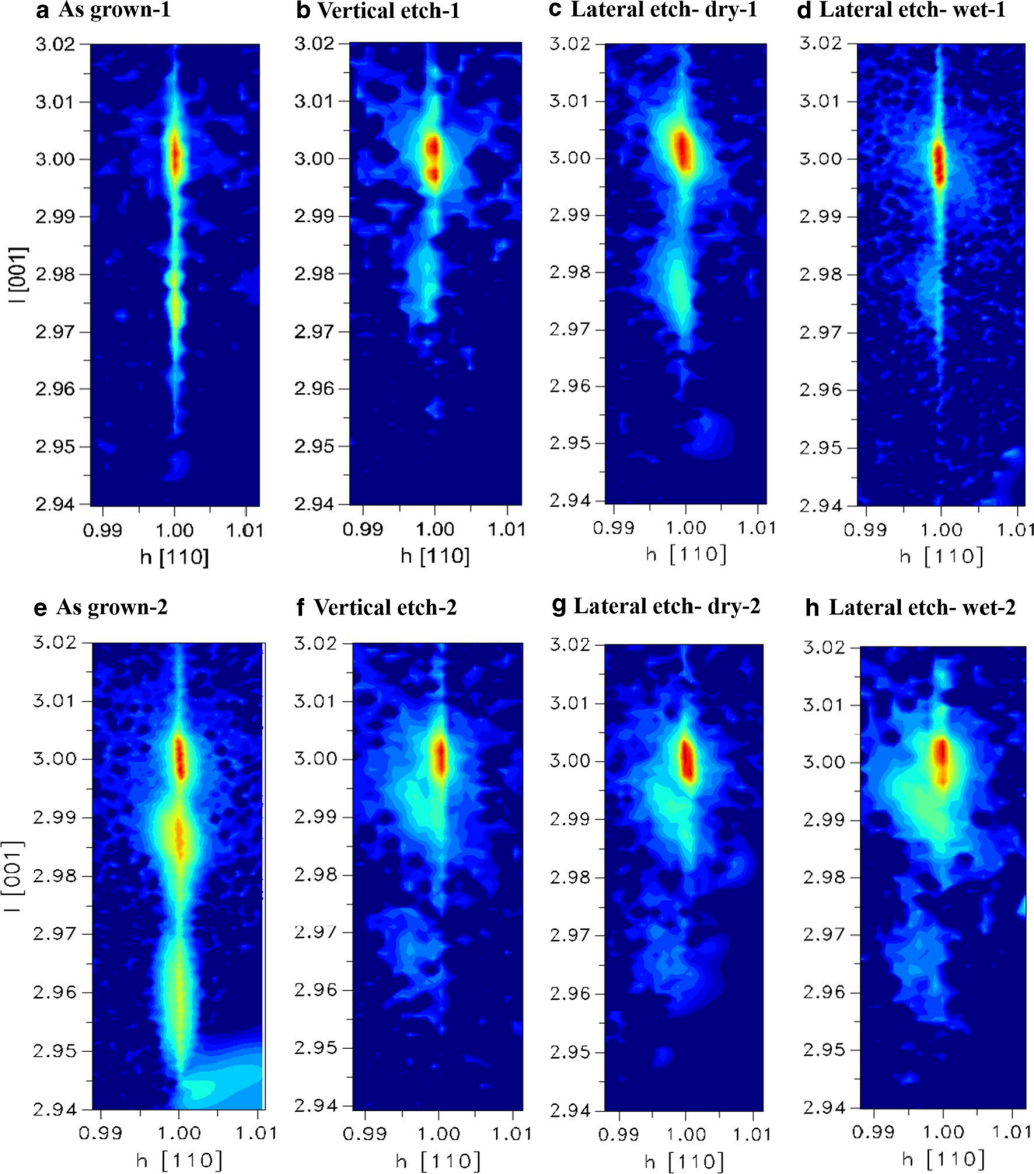
HRRLMs of P-doped Si/Si_0.86_Ge_0.14_/P-doped Si MLs with 5 nm spacer layer (sample-1) in **a**–**d**, and P-doped Si_0.93_Ge_0.07_/Si_0.78_Ge_0.22_/P-doped Si_0.93_Ge_0.07_ MLs (Sample-2) in **e**–**h**. The two mappings both have four panels: as grown, after vertical etch, lateral wet etch of HF (6%)/H_2_O_2_ (30%)/CH_3_COOH (99.8%) 20 min, and lateral dry etch of CF_4_/O_2_/He 11.5 s

Further X-ray analyses were performed to find out more information about the defect density in the samples in Fig. 9a–h. HRRLMs, which are based on two-dimensional measurements, were performed here as shown in Fig. 9a–h. The indicator for the defect density in HRRLMs is the broadening of SiGe layer along ω-direction (ω is the incident beam angle). The position of Si and SiGe peaks provides the strain components in-parallel and perpendicular to the growth direction. In sample-1 and sample-2, the as-grown SiGe layers show minor ω-broadening, and the layer is aligned to the Si showing fully-strained SiGe layers (see Fig. 9a, e). Figure 9b shows the sample after the vertical etch, the SiGe peak has shifted toward the Si substrate in a similar way in RC results in Fig. 8b indicating strain relaxation. But surprisingly, the lateral dry-etched sample (Fig. 9c) shows a clear ω-broadening of SiGe peak along with a shift in the reciprocal space, which is in direction out from the alignment with the Si peak. However, the wet-etched sample (in Fig. 9d) is fully-strain-aligned and has layer intensity lower than the dry-etched one (in Fig. 9c). In this case, it is expected that the generated defects have different origins in these samples since the nature of etch process is different. Sample-2 contains two SiGe layers; the Si_0.93_Ge_0.07_ peak is survived after etch in both vertical and lateral directions, while Si_0.78_Ge_0.22_ is disappeared after vertical etch showing full strain relaxation (Fig. 8f–h). The poor process stability of sample-2 could root from P-doping, which promote formation of misfit dislocations.

## Conclusions

In this work, the epitaxy of P-doped Si/SiGe/P-doped Si MLs along with etching of these MLs as initial structures for vGAAFET has been investigated. Firstly, the incorporation of P in Si/SiGe/Si MLs was studied. Different strategies for the epitaxy and ML structure have been proposed to eliminate the P-segregated peak at the interface of Si/SiGe heterostructure. From experiments, inserting undoped spacer layer could decrease the P peak. Hydrogen purge to clear the excess P atoms was not very helpful and stable P-P dimers could not be entirely removed. Substituting SiH_4_ with SiH_2_Cl_2_ as Si precursor to introduce Cl chemistry during the growth decreased the segregated P peak remarkably due to Cl active surface reactions. The impact of Si_0.93_Ge_0.07_ spacer layers after P-doped Si was also investigated. The results showed that the P peak at the SiGe interface disappeared, while the P incorporation in these layers improved by an order magnitude. In the second part of this study, the vertical etch of Si/SiGe/Si ML was performed to form NWs, and later, in these NWs, the SiGe was selectively wet- or dry-etched. The wet etching was sensitive to dopant concentration; meanwhile, dry etching was sensitive to Ge content. Dry etch was more appropriate for n-type structures with uniform etching profile and higher selectivity. For P-doped Si/Si_0.86_Ge_0.14_/P-doped Si MLs, the selectivity was 5.8 with dry etch and 4.2 for wet etch. The selectivity of P-doped Si_0.93_Ge_0.07_/Si_0.78_Ge_0.22_/P-doped Si_0.93_Ge_0.07_ MLs was 6.3 with dry etch and 2.5 with wet etch. The strain in SiGe was mostly preserved in Si/SiGe/Si after vertical and lateral etch; meanwhile, this strain in MLs with introduced Si_0.93_Ge_0.07_ spacer layer had poor stability after etch process.

## Data Availability

The authors declare that the data supporting the findings of this study are available within the article.

## Abbreviations

vGAAFET: Vertical gate-all-around field-effect transistors
ML: Multilayer
CMOS: Complementary metal oxide semiconductor
MBE: Molecular beam epitaxy
RPCVD: Reduced pressure chemical vapor deposition
RTCVD: Rapid thermal chemical vapor deposition
S/D: Source/drain
HRTEM: High-resolution transmission electron microscopy
HRXRD: High-resolution X-ray diffraction
HRRLM: High-resolution reciprocal lattice map
SEM: Scanning electron microscopy
SIMS: Secondary ion mass spectroscopy
RC: Rocking curve
FWHM: Full-width-half-maximum
HM: Hardmask
